# Metabolic profiling and pharmacological evaluation of alkaloids in three *Murraya* species

**DOI:** 10.3389/fpls.2025.1675533

**Published:** 2025-10-15

**Authors:** Huaxi Huang, Xiaoshan Geng, Lili Wang, Xuexue Wang, Fanglin Liu, Yude Peng, Chunfeng Tang, Rong Chen, Qin Liu

**Affiliations:** ^1^ Key Laboratory for Resource Plants Protection and Utilization of Yili Valley in Xinjiang, College of Biological Sciences and Technology, Yili Normal University, Yining, Xinjiang, China; ^2^ College of Agricultural Engineering, Guangxi Vocational University of Agriculture, Nanning, China; ^3^ National Center for TCM Inheritance and Innovation, Guangxi Botanical Garden of Medicinal Plants, Nanning, China

**Keywords:** *Murraya*, alkaloids, network pharmacology, tombozine, chemodiversity

## Abstract

This study comprehensively investigated the metabolic profiling, pharmacological potential, and biosynthetic regulation of alkaloids in three *Murraya* species (*M. exotica*, *M. kwangsiensis*, and *M. tetramera*). Through integrative multi-omics approaches, including metabolomics, transcriptomics, network pharmacology, and molecular docking, a total of 77 alkaloids were identified, categorized into 18 structural classes. Comparative analysis revealed species-specific accumulation patterns, with 50 alkaloids shared among all three species and unique metabolites detected in *M. exotica* and *M. kwangsiensis*. Principal component analysis (PCA) confirmed distinct alkaloid profiles, highlighting interspecies divergence. Network pharmacology identified 427 potential targets for 12 bioactive alkaloids, with core targets (PIK3CA, PIK3CD, MAPK8, and JAK2) implicated in cancer-related pathways such as PI3K-Akt signaling. Molecular docking demonstrated strong binding affinities between key alkaloids (tombozine, aegeline, and crotaleschenine) and oncogenic targets, suggesting antitumor mechanisms via modulation of proliferation and apoptosis. Transcriptomic analysis elucidated the biosynthetic pathway of tombozine, linking differential gene expression (DDC/TDC homologs) to species-specific alkaloid accumulation. These findings underscore the pharmacological diversity of *Murraya* alkaloids and provide a foundation for targeted drug development and sustainable utilization of medicinal plant resources.

## Introduction

Plants of the genus *Murraya* (Rutaceae) are evergreen shrubs or small trees native to southern China and various tropical/subtropical regions of Asia (Yohanes et al., 2023). Renowned for their distinctive fragrance and medicinal value, these plants have been extensively utilized as traditional herbal remedies throughout history. Characterized by a pungent, slightly bitter taste and warm nature, they exhibit therapeutic effects including qi-activation for pain relief, blood stasis resolution, and detoxification, making them valuable for treating epigastric pain, rheumatic arthralgia, and venomous snake bites (Balakrishnan et al., 2020; Liu et al., 2020). Modern pharmacological studies have revealed that *Murraya* species contain complex phytochemical compositions, primarily coumarins, flavonoids, alkaloids, and volatile oils, which collectively confer diverse bioactivities (Khalid et al., 2023; You et al., 2021). Particularly notable are the alkaloid metabolites, which have emerged as research priorities due to their remarkable pharmacological properties including anti-inflammatory, analgesic, antimicrobial, and antitumor effects, constituting valuable lead compounds for novel drug development (Zhou et al., 2024).

Alkaloids, a class of nitrogen-containing organic compounds ubiquitous in plants, play pivotal roles in both ecological functions and pharmaceutical applications. In plant systems, these specialized metabolites serve as chemical defenses against herbivores and pathogens by producing repellent odors or toxic substances (Wu et al., 2023). Moreover, they participate in key physiological processes including signal transduction regulation and growth rhythm modulation (Ahmad et al., 2024). From a pharmaceutical perspective, alkaloids demonstrate remarkable medicinal potential through diverse bioactivities, serving as valuable resources for drug discovery (Nett et al., 2020). Notably, over 30% of clinically used drugs derive from alkaloid compounds, as exemplified by morphine and codeine with potent analgesic effects (Vadhel et al., 2023). Other medicinally significant alkaloids include isoquinoline alkaloids in *Corydalis* species exhibiting multi-target bioactivities in cardiovascular and neurological therapeutics (Ma et al., 2008), and lycorine alkaloids in *Lycoris* species demonstrating tumor-suppressive effects (Cahlíková et al., 2020). Despite their therapeutic potential, research progress faces substantial challenges due to structural complexity, incomplete biosynthetic pathway characterization, and poorly understood metabolic regulation networks (Song et al., 2022). The genus *Murraya* encompasses three pharmacologically significant species with distinct ecological and phytochemical profiles: *M. exotica*, widely distributed in southern China and officially listed in the Chinese Pharmacopoeia (Zhou and Fu, 2021); *M. tetramera*, predominantly found in Yunnan and Guangxi regions serving as an ethnomedicinal resource (Huang et al., 2025); and *M. kwangsiensis*, an endemic species exclusive to Guangxi (Huang et al., 2025). Comparative studies on their alkaloid compositions are crucial for elucidating the genus’ chemical diversity and establishing scientific foundations for sustainable utilization. However, current research remains limited by insufficient understanding of their biosynthetic pathways and regulatory mechanisms, particularly for the less-studied *M. kwangsiensis* and *M. tetramera*.

Therefore, this study aims to systematically investigate and compare the alkaloid profiles of three understudied *Murraya* species (*M. exotica*, *M. kwangsiensis*, and *M. tetramera*). By integrating multi-omics and computational approaches, we seek to decipher their pharmacological potential and biosynthetic mechanisms. This research not only provides a scientific basis for the sustainable utilization of these medicinal resources but also establishes a translational model for bridging traditional ethnopharmacology with modern functional genomics.

## Materials and methods

### Plant materials

Experimental materials were collected from the medicinal plant nursery (E108°22’, N22°51’) in Guangxi Zhuang Autonomous Region. Specifically, three *Murraya* species - *Murraya exotica* (Me), *Murraya tetramera* (Mt), *and Murraya kwangsiensis* (Mk) - were selected as study subjects. For each species, nine distinct individual plants with comparable growth vigor and morphological characteristics were selected. The nine plants from each species were then grouped into three biological replicates (n=3 per species), with each replicate consisting of a pooled sample of leaves from three individual plants. Leaves were harvested from identical positions of 1-year-old branches. To ensure material integrity for omics analysis, only healthy leaves showing no signs of disease, pest infestation, or physical damage were selected. Using tools sterilized with 75% ethanol, leaves were finely sectioned and immediately transferred to sterile cryovials. Immediately following collection, all samples were flash-frozen in liquid nitrogen for downstream analyses.

### Alkaloid metabolites profiling

Samples were vacuum freeze-dried and precisely weighed (50 mg) prior to homogenization with 1000 μL extraction solvent (methanol: acetonitrile: water = 1:2:1, v/v/v) using 30-second vortex mixing. Subsequently, stainless steel beads were added for mechanical disruption at 45 Hz (10 min) followed by ice-bath ultrasonication (10 min). Following this, the homogenates underwent phase separation at -20 °C for 1 h. Centrifugation was then performed at 12,000 rpm (4 °C, 15 min) to pellet insoluble debris. Finally, 300 μL supernatant from each sample was filtered through 0.22 μm organic membranes into 2 mL injection vials. A pooled quality control (QC) sample was prepared by combining 10 μL aliquots from individual extracts for instrumental analysis.

Chromatographic separation was performed on a UPLC-ESI-MS/MS system (UPLC: Waters Acquity I-Class PLUS; MS: Applied Biosystems QTRAP 6500^+^) equipped with a Waters HSS-T3 column (1.8 µm, 2.1 × 100 mm). The mobile phase consisted of solvent A (ultrapure water containing 0.1% formic acid and 5 mM ammonium acetate) and solvent B (acetonitrile with 0.1% formic acid). Specifically, the gradient elution program was optimized as follows: initial conditions of 98% A/2% B held for 1.5 min, followed by a linear gradient to 50% A/50% B over 5.0 min, then to 2% A/98% B over 9.0 min. The latter ratio was maintained for 1 min before re-equilibrating to initial conditions (98% A/2% B) within 1 min and holding for 3 min. Throughout the analysis, the flow rate remained constant at 0.35 mL/min with column temperature stabilized at 50 °C. Aliquots (4 μL) were injected, and column effluent was directed to an ESI-triple quadrupole-linear ion trap (QTRAP) mass spectrometer via alternating connections.

Electrospray ionization (ESI) was operated at 550 °C with ion spray voltages of 5,500 V (positive mode) and -4,500 V (negative mode). Specifically, gas flow rates were optimized as follows: ion source gas I at 50 psi, gas II at 55 psi, and curtain gas at 35 psi, while collision-induced dissociation was set to medium. Prior to analysis, instrument tuning and mass calibration were performed using 10 μmol/L (QQQ mode) and 100 μmol/L (LIT mode) polypropylene glycol solutions, respectively. Multiple reaction monitoring (MRM) scans were conducted in QQQ mode with collision gas (nitrogen) maintained at medium pressure. Subsequently, declustering potential and collision energy for individual MRM transitions were systematically optimized through iterative parameter adjustments. Throughout the runs, specific MRM ion pairs were dynamically monitored based on metabolite elution profiles within each acquisition cycle.

Metabolite identification was conducted using our in-house GB-PLANT database through secondary spectral matching. Specifically, isotopic signals and redundant adducts (including K^+^, Na^+^, and NH_4_^+^, ions) were systematically excluded during analysis. Additionally, fragment ions derived from higher molecular weight compounds were filtered to eliminate interference. Quantitative analysis was performed via multiple reaction monitoring (MRM) mode on a triple quadrupole mass spectrometer. In this workflow, the first quadrupole selectively isolated target precursor ions, effectively excluding isobaric interferences. Subsequently, the collision cell fragmented these ions, generating characteristic product ions that were further filtered by the third quadrupole to ensure analytical specificity. Following ion selection, chromatographic peak areas were integrated across all samples. To enhance reproducibility, intra-batch correction was applied to align peak integration parameters for identical metabolites detected in different sample runs.

Differential metabolites were screened using the criteria of |fold change| ≥ 1.0, VIP (variable importance in projection) ≥ 1, and a t-test *p*-value < 0.05. Identified metabolites were annotated using the KEGG (Kyoto Encyclopedia of Genes and Genomes) compound database (http://www.kegg.jp/kegg/compound/). Subsequently, these annotated metabolites were mapped to the KEGG pathway database ((http://www.kegg.jp/kegg/pathway.html).

### RNA-sequencing

RNA sequencing and library construction were performed by Biomarker Technologies Co., Ltd (Beijing, China). Specifically, total RNA was isolated from leaf tissues using TRIzol reagent. RNA purity and concentration were assessed using a NanoDrop 2000 spectrophotometer, while integrity was evaluated with an Agilent 2100 Bioanalyzer (RNA Integrity Number ≥8.0). Following rigorous quality control, qualified RNA samples were processed for transcriptome analysis.

Following successful RNA qualification, cDNA library preparation was performed through the following workflow: Eukaryotic mRNA was isolated using Oligo(dT)-coated magnetic beads. Purified mRNA was randomly fragmented in Fragmentation Buffer. First- and second-strand cDNA were sequentially synthesized, followed by purification using AMPure XP beads. Double-stranded cDNA underwent end repair, poly-A tailing, and adapter ligation, after which fragments of 300–500 bp were selectively retained through bead-based size selection. Final libraries were enriched via 15-cycle PCR amplification. Following library assembly, preliminary quantification was conducted using a Qubit 3.0 Fluorometer (concentrations exceeding 1 ng/μL). Insert size distribution was then verified on a Qsep400 Bio-Fragment Analyzer. Finally, library effectiveness was validated through quantitative PCR (qPCR) with a threshold of >2 nM. Qualified libraries were sequenced on an Illumina platform using paired-end 150 bp (PE150) chemistry.

Functional annotation of Unigenes was systematically performed through multi-database alignment. First, nucleotide sequences were aligned against NR, Swiss-Prot, COG, KOG, eggNOG 4.5, and KEGG databases using DIAMOND software. Subsequently, KEGG Orthology (KO) assignments were generated through KOBAS analysis, while GO annotations were obtained via InterProScan using InterPro-integrated databases. Following amino acid sequence prediction, Pfam domain identification was conducted with HMMER. For differential expression analysis, read counts were normalized across samples prior to applying DESeq2 for comparisons with biological replicates. Stringent thresholds of absolute fold change ≥2 and false discovery rate (FDR) <0.01 were employed to identify significantly differentially expressed genes (DEGs).

### Pharmacological efficiency analysis of alkaloid metabolites

Based on the alkaloid metabolites identified in *Murraya* species through metabolomics, pharmacologically active alkaloids and their associated disease information were retrieved from the PubChem database (https://pubchem.ncbi.nlm.nih.gov/). The Simplified Molecular Input Line Entry System (SMILES) files of these metabolites were also acquired from PubChem. Potential therapeutic targets of these alkaloids were predicted using the Swiss Target Prediction database (http://www.swisstargetprediction.ch/) under *Homo sapiens* species constraints, with a probability threshold set to > 0.1. Subsequently, a metabolite-target interaction network was constructed and visualized using Cytoscape 3.9.1 software.

Protein-protein interaction (PPI) analysis of the predicted targets was conducted using the STRING 12.0 database (https://cn.string-db.org/) with the following parameters: species limited to *Homo sapiens* and a high-confidence interaction score threshold (> 0.9). Overlapping targets were further analyzed using the cytoHubba plugin in Cytoscape 3.9.1. Core targets were identified by selecting nodes with Maximum Clique Centrality (MCC) values exceeding their respective means. A PPI network of these core targets was subsequently generated.

The identified core targets were subjected to Gene Ontology (GO) enrichment analysis (biological processes, cellular components, and molecular functions) and Kyoto Encyclopedia of Genes and Genomes (KEGG) pathway enrichment analysis using the STRING 12.0 database. Visualization of the results was performed using R version 4.2.0, adhering to standardized analytical workflows.

Additionally, disease-related targets of the pharmacologically active alkaloids were retrieved from the GeneCards database (https://www.genecards.org/) using a relevance score threshold of > 5. Duplicate entries were removed, and the remaining targets underwent normalization. Finally, an integrated network diagram illustrating interactions among alkaloids, targets, pathways, and diseases was constructed using Cytoscape 3.9.1 software.

### Molecular docking

The top five core targets were identified as key candidates through the Maximum Clique Centrality (MCC) scoring method. The three-dimensional (3D) structures of these key target proteins were retrieved from the UniProt database (https://www.uniprot.org/), while the 3D structures of candidate metabolites were obtained from the PubChem database (https://pubchem.ncbi.nlm.nih.gov/). Subsequently, molecular docking simulations were performed using the CB-DOCK2 platform (https://cadd.labshare.cn/cb-dock2/index.php) to evaluate binding interactions and visualized using Discovery Studio 2019.

### Data statistics

The experimental data were analyzed using Microsoft Office 2021 (Microsoft Corporation, Redmond, WA, USA). VIP diagrams, classification pie charts, principal component analysis (PCA), and Venn diagrams were generated through R version 3.5.1. However, the Enrichment_Bubble_Plot was specifically implemented in Python 3.6.6. Furthermore, complementary analytical representations comprising heatmaps and comparative Venn diagrams were constructed via TBtools-II v2.154 (Chen et al., 2023).

## Results

### Comprehensive alkaloids profiling

**Figure 1:**
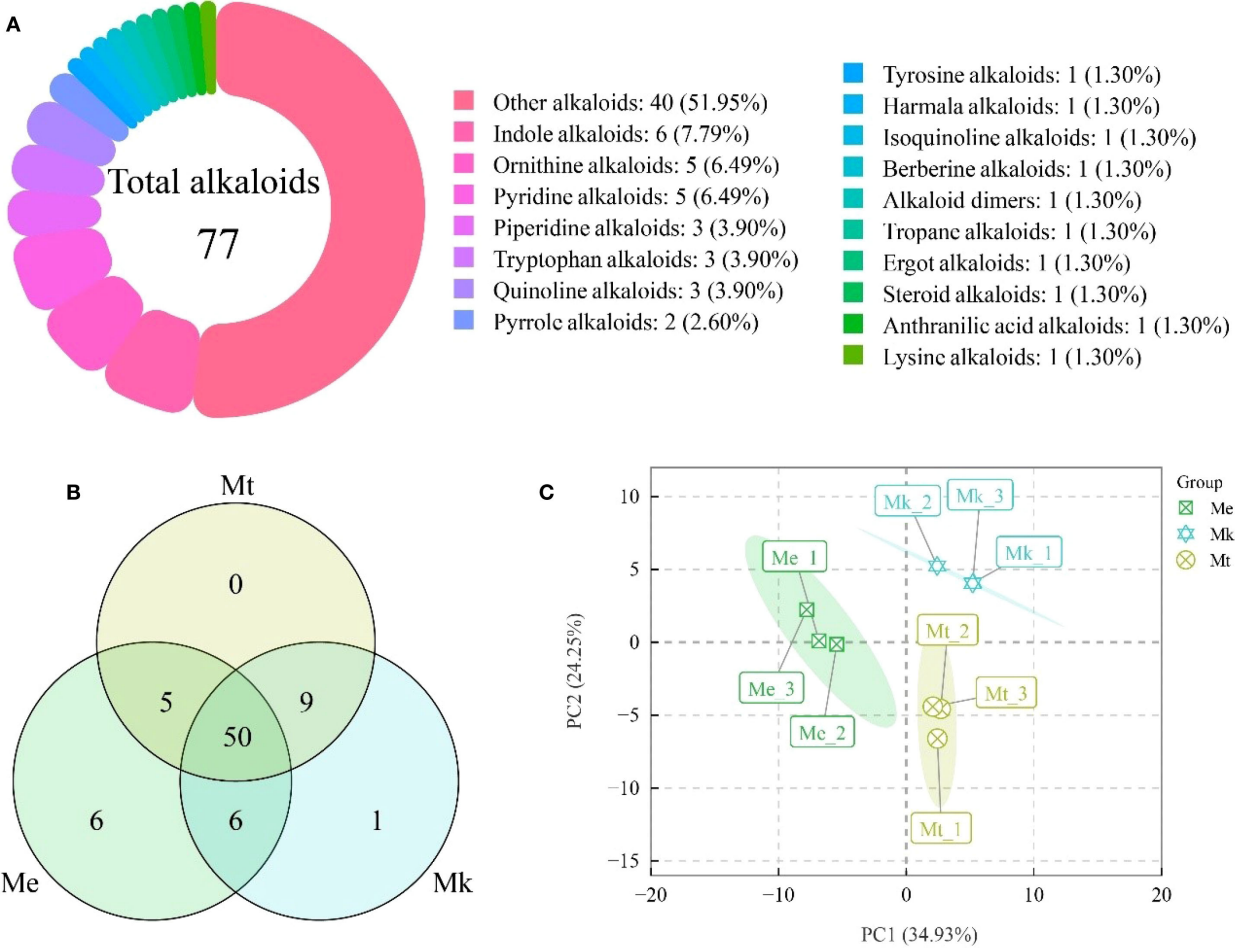
Integrated analysis of alkaloid metabolites in three *Murraya* species. **(A)** Classification of identified alkaloids. **(B)** Venn diagram of alkaloid profiles across the three *Murraya* species. **(C)** Principal component analysis (PCA) score plot derived from alkaloid composition data. Me, *Murraya exotica*; Mt, *Murraya tetramera*; Mk, *Murraya kwangsiensis*.

Metabolomic analysis of three *Murraya* species (*M. exotica* [Me], *M. kwangsiensis* [Mk], and *M. tetramera* [Mt]) identified 77 alkaloids (**Figure 1A**), categorized into 18 structural classes: Alkaloid dimers, anthranilic acid alkaloids, berberine alkaloids, ergot alkaloids, harmala alkaloids, indole alkaloids, isoquinoline alkaloids, lysine alkaloids, ornithine alkaloids, other alkaloids, piperidine alkaloids, pyridine alkaloids, pyrrole alkaloids, quinoline alkaloids, steroid alkaloids, tropane alkaloids, tryptophan alkaloids, and tyrosine alkaloids. Notably, 51.95% of alkaloids were classified as other alkaloids, while the remaining 17 categories each contained fewer than 10 metabolites.

Comparative analysis revealed conserved alkaloid diversity across accessions (**Figure 1B**). Me, Mk, and Mt contained 67, 66, and 64 alkaloids, respectively, with 50 shared across all three. Intriguingly, 6 and 1 unique alkaloids were detected exclusively in Me and Mk, respectively, whereas Mt exhibited no species-specific metabolites.

Principal component analysis (PCA) of alkaloid profiles explained 59.18% of total variance (PC1: 34.93%; PC2: 24.25%) (**Figure 1C**; **Supplementary Table S1**). Strikingly, triplicate samples clustered tightly within each accession, confirming experimental reproducibility and reliability. Furthermore, distinct segregation of the three accessions highlighted divergence in their alkaloid accumulation patterns.

### Differential alkaloid accumulation patterns

**Figure 2:**
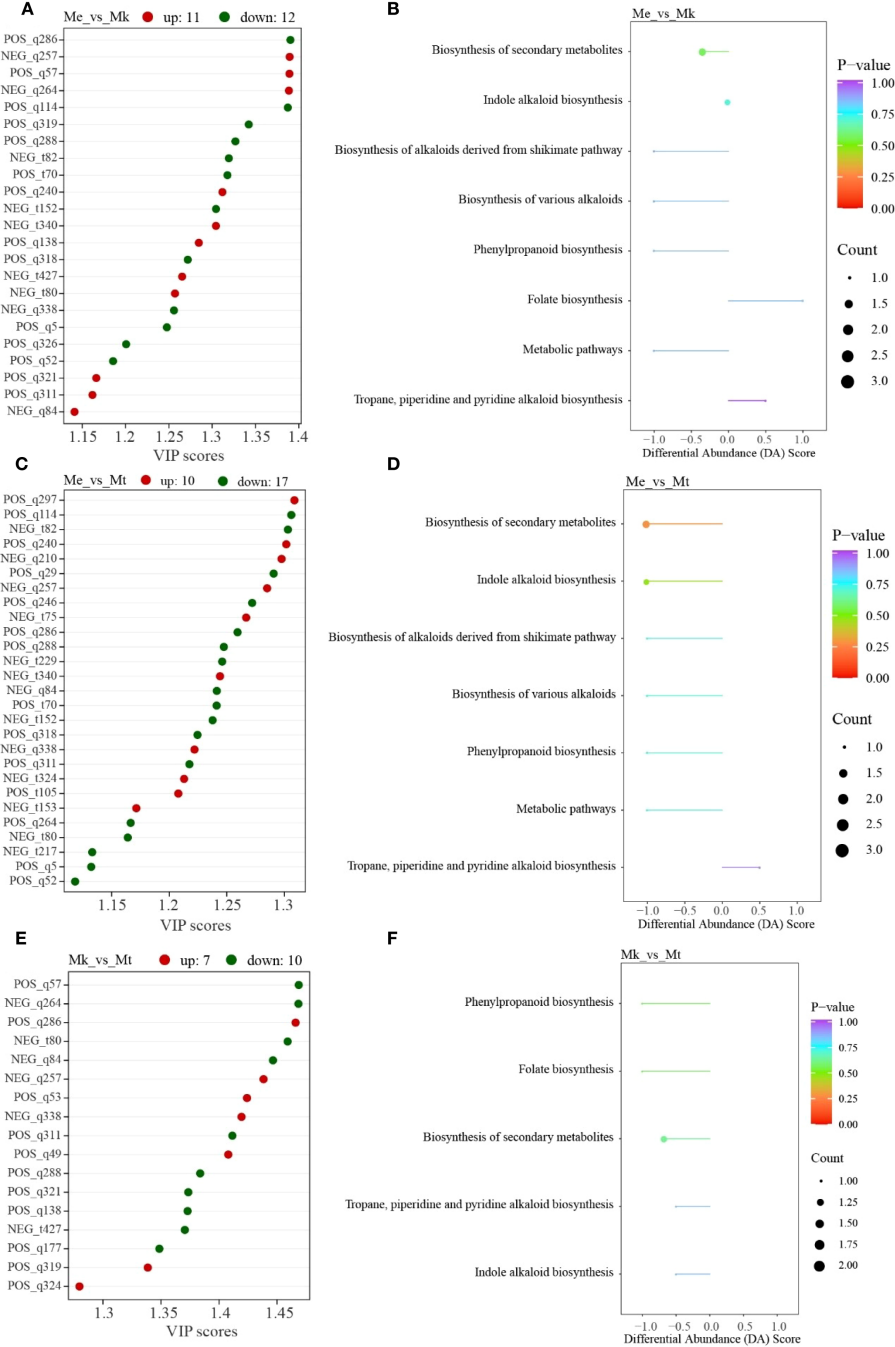
Integrated analysis of differential alkaloid metabolites in three *Murraya* species. **(A)** VIP score plot of differential accumulated metabolites (DAMs) in Me_vs_Mk comparison group. **(B)** Corresponding KEGG pathway enrichment analysis of DAMs from the Me_vs_Mk comparison group. **(C)** VIP score distribution of DAMs in Me_vs_Mt comparison group. **(D)** KEGG enrichment profiles of DAMs identified in the Me_vs_Mt comparison group. **(E)** Comparative VIP scores of DAMs between Mk_vs_Mt comparison group. **(F)** Pathway enrichment mapping of Mk_vs_Mt DAMs based on KEGG ontology. Me, *Murraya exotica*; Mt, *Murraya tetramera*; Mk, *Murraya kwangsiensis*.

A total of 38 differential alkaloid metabolites (DAMs) were identified across three *Murraya* species based on predefined thresholds. Twenty-three DAAMs were detected in Me_vs_Mk comparison group (**Figure 2A**), comprising 11 upaccumulated and 12 downaccumulated metabolites in Mk relative to Me. KEGG enrichment analysis revealed eight significantly enriched pathways (**Figure 2B**), including: Folate biosynthesis, Biosynthesis of various alkaloids, Biosynthesis of alkaloids derived from shikimate pathway, Phenylpropanoid biosynthesis, Biosynthesis of secondary metabolites, Indole alkaloid biosynthesis, Tropane, piperidine and pyridine alkaloid biosynthesis, and Metabolic pathways.

Twenty-seven DAMs were identified in Me_vs_Mt comparison group (**Figure 2C**), with 10 upaccumulated and 17 downaccumulated in Mt versus Me. Seven pathways showed significant enrichment (**Figure 2D**): Biosynthesis of various alkaloids, Biosynthesis of alkaloids derived from shikimate pathway, Phenylpropanoid biosynthesis, Biosynthesis of secondary metabolites, Indole alkaloid biosynthesis, Metabolic pathways, Tropane, piperidine and pyridine alkaloid biosynthesis.

Seventeen DAMs were observed in Mk_vs_Mt comparison group (**Figure 2E**), exhibiting 7 upaccumulated and 10 downaccumulated metabolites in Mt compared to Mk. Five pathways were significantly enriched (**Figure 2F**): Folate biosynthesis, Phenylpropanoid biosynthesis, Biosynthesis of secondary metabolites, Indole alkaloid biosynthesis, Tropane, piperidine and pyridine alkaloid biosynthesis.

Intersection analysis (**Supplementary Figure S1**) identified seven core DAMs shared across all comparisons. Notably, the Mk_vs_Mt and Me_vs_Mt comparisons showed no overlapping metabolites, while the Me_vs_Mt comparison group contained 11 unique DAMs. Collectively, these findings demonstrate species-specific alkaloid accumulation profiles among the three *Murraya* species.

### Analysis of potential targets

**Figure 3:**
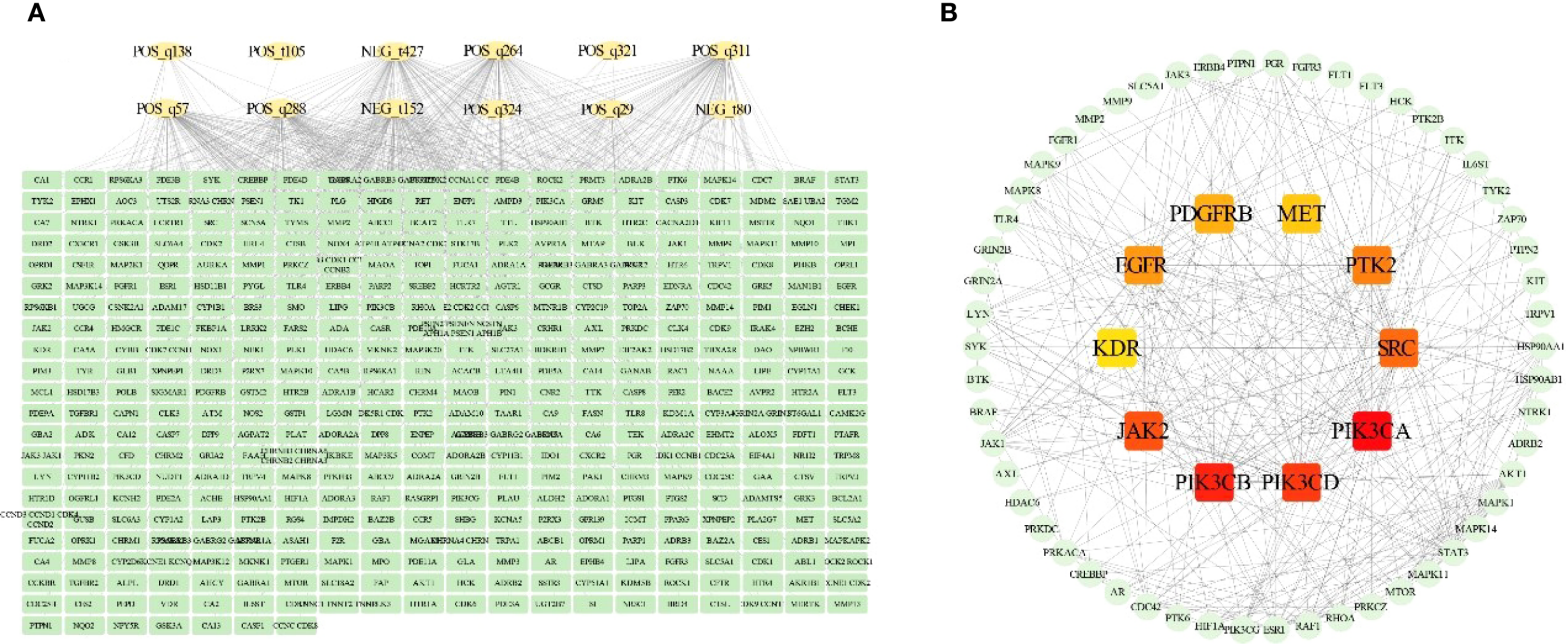
Integrated analysis of potential targets and alkaloid metabolites. **(A)** Interaction network between alkaloid metabolites and potential targets. **(B)** Protein-protein interaction (PPI) network of potential targets.

We analyzed potential targets for 38 differential alkaloid metabolites, identifying 427 potential interaction targets associated with 12 bioactive metabolites. Construction of their regulatory network elucidated the relationships between differential alkaloid metabolites and potential targets (**Figure 3A**). To further screen candidate core targets, we conducted protein-protein interaction (PPI) network analysis on the identified targets (**Figure 3B**). Using maximal clique centrality (MCC) ranking, we identified 64 candidate core targets. Notably, 10 targets occupied central positions in the PPI network topology: PIK3CA (Phosphatidylinositol 4,5-bisphosphate 3-kinase catalytic subunit alpha isoform), PIK3CB (Phosphatidylinositol 4,5-bisphosphate 3-kinase catalytic subunit beta isoform), PIK3CD (Phosphatidylinositol 4,5-bisphosphate 3-kinase catalytic subunit delta isoform), JAK2 (Tyrosine-protein kinase JAK2), SRC (Proto-oncogene tyrosine-protein kinase Src), PTK2 (Focal adhesion kinase 1), EGFR (Epidermal growth factor receptor), PDGFRB (Platelet-derived growth factor receptor beta), MET (Hepatocyte growth factor receptor), and KDR (Vascular endothelial growth factor receptor 2). These pivotal targets may play crucial roles in mediating the pharmacological effects of alkaloid metabolites.

### Enrichment analysis of candidate targets

**Figure 4:**
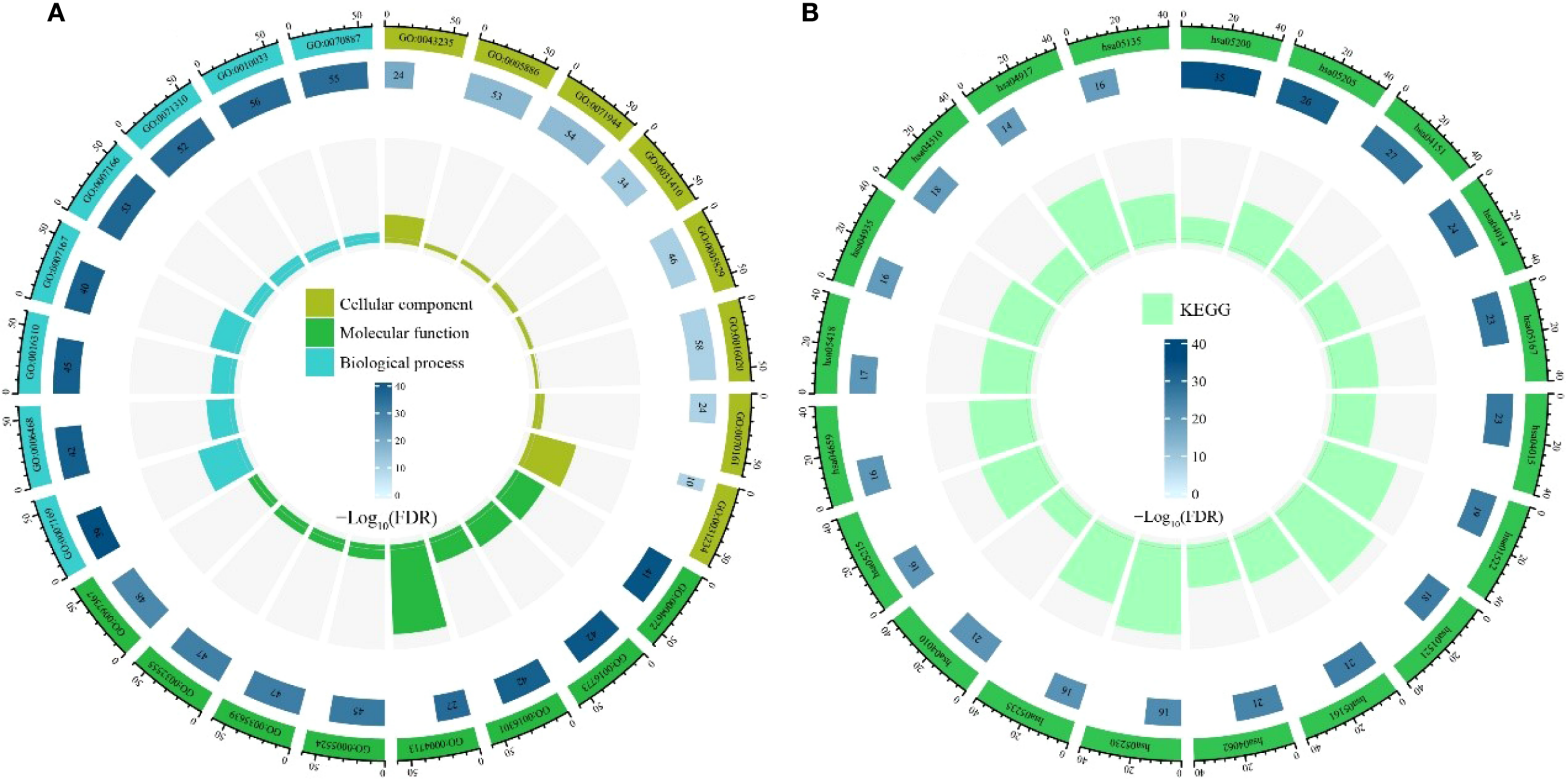
Enrichment analysis of candidate targets. **(A)** Gene Ontology (GO) enrichment analysis. **(B)** Kyoto Encyclopedia of Genes and Genomes (KEGG) pathway enrichment analysis.

GO enrichment analysis of the 64 candidate core targets revealed multifaceted pharmacological mechanisms mediating alkaloid metabolites’ therapeutic effects (**Figure 4A**). Notably, cellular component enrichment in plasma membrane-associated structures (e.g., receptor complexes, anchoring junctions) underscores their role in modulating extracellular signal perception and intercellular communication, a critical feature for drug candidates targeting membrane-bound receptors in cancer or inflammatory disorders. Molecular function clustering, predominantly kinase-related activities (phosphotransferase activity, protein tyrosine kinase activity) and nucleotide binding (ATP binding, purine ribonucleotide binding), directly correlates with enzymatic regulation of signaling cascades, suggesting alkaloids may disrupt phosphorylation-dependent processes or energy metabolism critical to proliferation and apoptosis. Biological process enrichment, particularly in transmembrane receptor-mediated signaling (e.g., EGFR, MET) and stress-responsive pathways, implies dual regulatory capacities: modulating growth factor signaling while enhancing cellular adaptation to biochemical stressors, mechanisms consistent with neuroprotective and chemopreventive properties. Collectively, these findings establish alkaloid-target interactions as pivotal modulators of signal transduction networks, providing mechanistic insights into their documented anti-inflammatory, antitumor, and homeostatic regulatory effects.

KEGG enrichment analysis of the 64 candidate core targets demonstrated their significant involvement in cancer pathogenesis and therapeutic resistance mechanisms, underscoring the multitarget pharmacological potential of alkaloids (**Figure 4B**). Notably, enrichment in Pathways in Cancer and the integrated PI3K-Akt/Ras/MAPK signaling axis highlights alkaloids’ ability to regulate oncogenic proliferation, apoptosis evasion, and metastasis, key mechanisms for chemopreventive strategies. The co-enrichment of EGFR tyrosine kinase inhibitor resistance and the PD-1/PD-L1 checkpoint pathway indicates dual therapeutic benefits: overcoming drug resistance while enhancing immune checkpoint blockade efficacy. Furthermore, enrichment in Th17 cell differentiation and chemokine signaling pathways suggests immunomodulatory properties that may counteract inflammation-associated carcinogenesis. The prominence of focal adhesion and proteoglycans in cancer pathways further implicates alkaloids in disrupting tumor microenvironment remodeling and angiogenesis. Crucially, endocrine resistance- and central carbon metabolism-associated pathways correlate with inhibition of metabolic reprogramming, a recognized feature of anticancer alkaloids. Collectively, these interactions establish the core targets as pivotal regulators of oncogenic signaling networks, enhancing chemosensitivity and immune surveillance, fundamental mechanisms for developing alkaloid-based therapies against diverse malignancies, including drug-resistant cancers and inflammatory disorders.

### Integrated network analysis of metabolite-target-pathway-disease interactions

**Figure 5:**
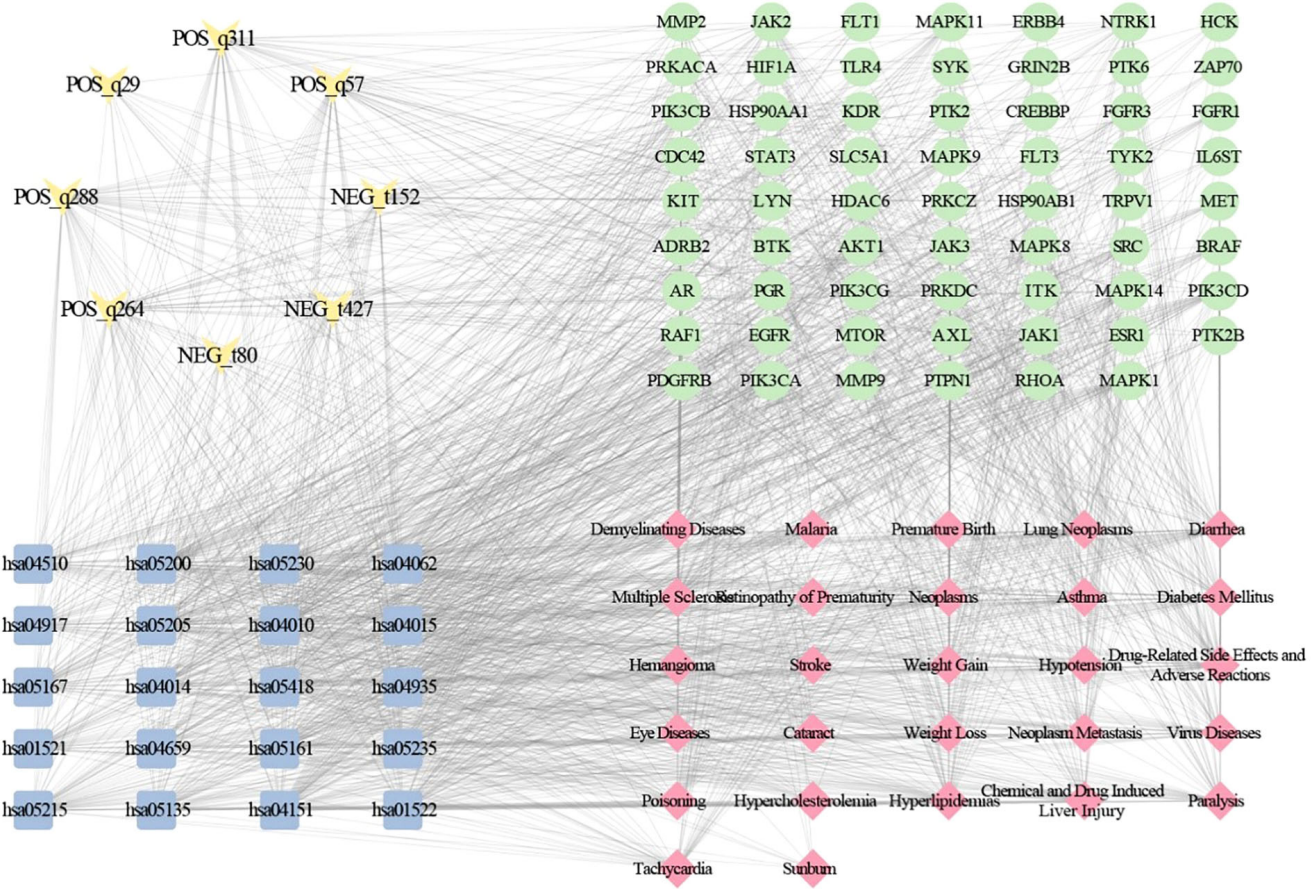
Mechanistic regulatory network mapping of metabolite-target-pathway-disease crosstalk.

To establish metabolite-disease correlations, we analyzed 12 metabolites with potential targets, revealing that 8 metabolites exhibited associations with 27 distinct diseases and 20 significantly enriched KEGG pathways (**Figure 5**). Using the MCC algorithm, we identified five pivotal alkaloid metabolites, POS_q288 (sinapine), POS_q264 (piperlongumine), POS_q57 (aegeline), POS_q311 (tombozine), and NEG_t427 (crotaleschenine) that demonstrated strong linkages to four core targets (PIK3CD, MAPK8, PIK3CA, and JAK2), five disease categories (drug-related side effects and adverse reactions, neoplasms, neoplasm metastasis, multiple sclerosis, and demyelinating diseases), and five cancer-related pathways: hsa05200 (pathways in cancer), hsa04151 (PI3K-Akt signaling), hsa05167 (Kaposi sarcoma-associated herpesvirus infection), hsa05161 (hepatitis B), and hsa05205 (proteoglycans in cancer). Notably, both disease and pathway associations predominantly converged on neoplasms-related mechanisms, highlighting the critical role of *Murraya* alkaloids in neoplasms pathophysiology.

### Molecular docking and evaluation of *Murraya* species

**Figure 6:**
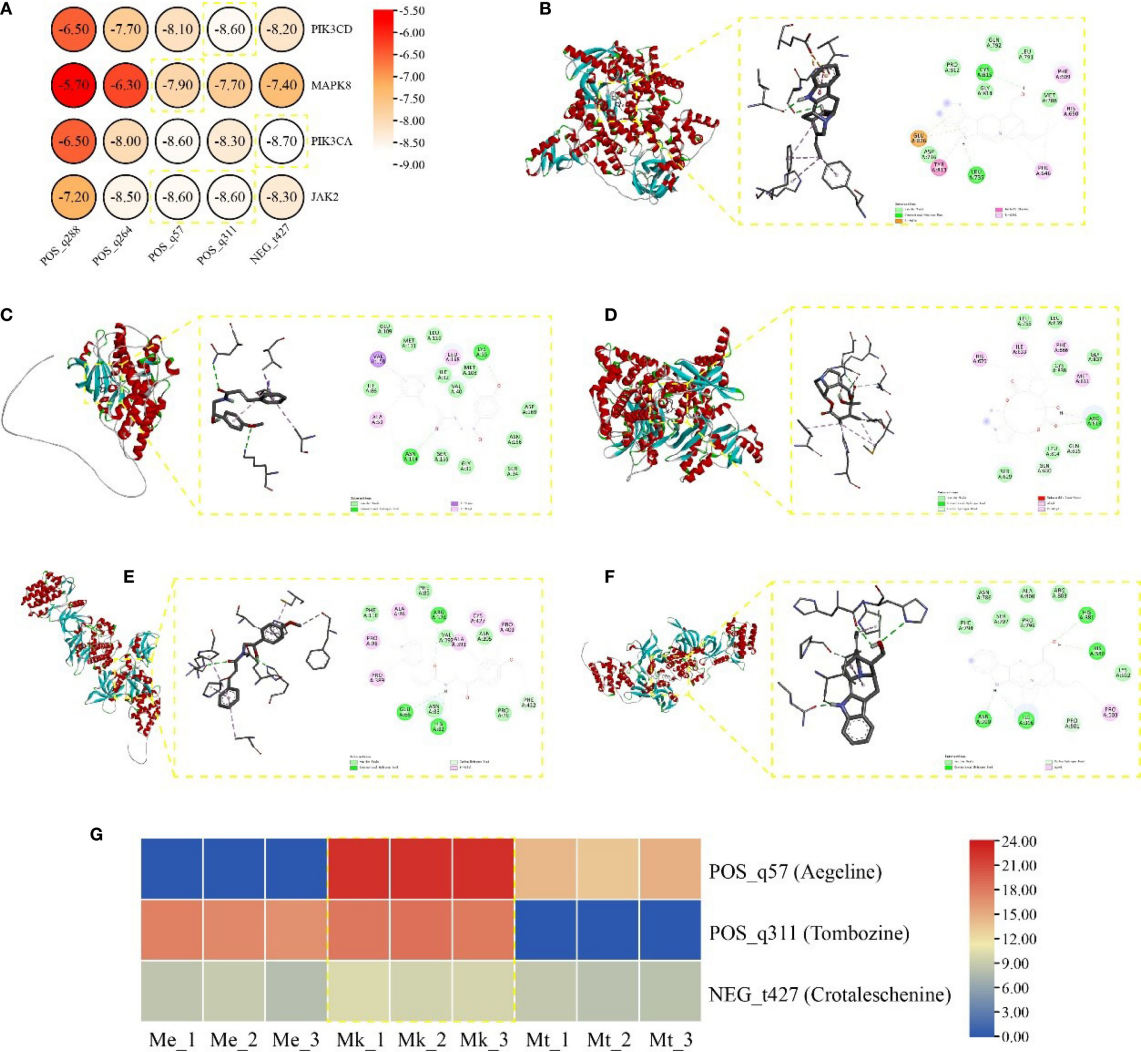
**(A)** Heatmap of binding energy. Molecular docking conformation of **(B)** PIK3CD with N- Tombozine **(B)**, MAPK8 with Aegeline **(C)**, PIK3CA with Crotaleschenine **(D)**, **(E)** JAK2 with Aegeline **(E)**, JAK2 with Tombozine **(F)**. **(G)** Heatmap of pharmacological efficacy alkaloids in *Murraya*.

Molecular docking analysis was performed between five key alkaloid metabolites and four core targets, with binding affinities visualized via heatmap (**Figure 6A**). All calculated binding energies were below -5 kcal/mol, indicating thermodynamically favorable ligand-receptor interactions.

Notably, POS_q311 (Tombozine) exhibited the strongest binding to PIK3CD (-8.60 kcal/mol), primarily mediated through van der waals, amide-pi stacked, pi-anion, conventional hydrogen bond, and pi-alkyl (**Figure 6B**). POS_q57 (Aegeline) demonstrated optimal stability with MAPK8 (-7.90 kcal/mol), stabilized by van der waals, conventional hydrogen bond, pi-alkyl, and pi-sigma (**Figure 6C**). NEG_t427 (Crotaleschenine) showed the highest binding affinity for PIK3CA (-8.70 kcal/mol), involving van der waals, conventional hydrogen bond, carbon hydrogen bond, pi-alkyl, alkyl, and unfavorable donor-donor (**Figure 6D**). Interestingly, both POS_q57 and POS_q311 displayed equivalent binding energies (-8.60 kcal/mol) with JAK2, stabilized through van der waals, conventional hydrogen bond, carbon hydrogen bond, and pi-alkyl. (**Figures 6E, F**). These results collectively identified van der Waals forces, pi-alkyl interactions, and conventional hydrogen bonds as critical contributors to ligand-receptor complex stability.

Further analysis of accumulation patterns revealed that the three top-performing metabolites showed predominant accumulation in Mk compared to other materials (**Figure 6G**), suggesting Mk’s enhanced potential for medicinal applications.

### Metabolic pathway analysis

**Figure 7:**
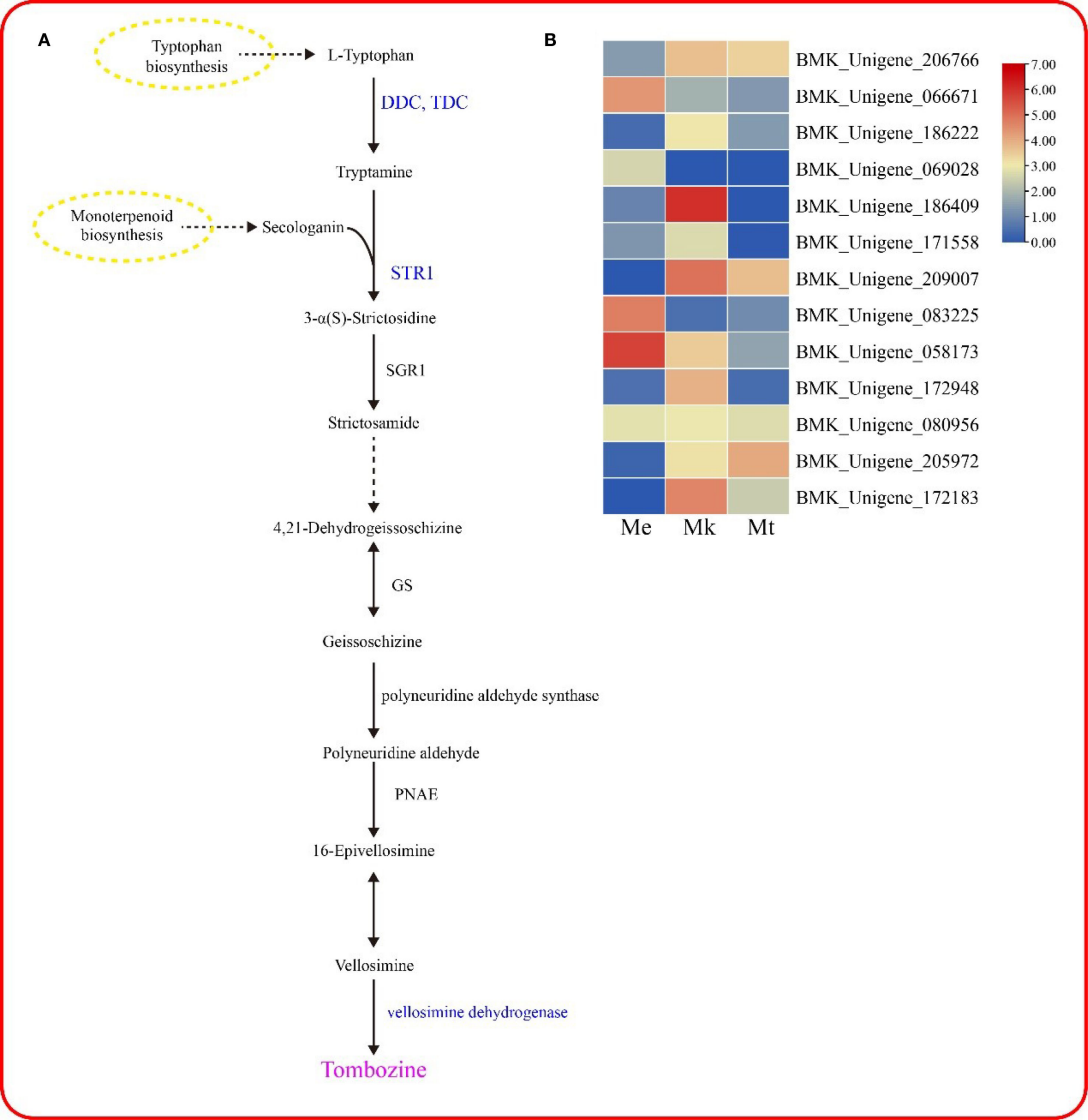
Metabolic pathway analysis. **(A)** Biosynthetic pathway of tombozine in *Murraya* plants. **(B)** Differentially expressed genes (DEGs) within the metabolic pathway.

Tombozine is synthesized via the indole alkaloid biosynthesis pathway, initiating from L-tryptophan as the primary substrate (**Figure 7A**). Under the catalytic action of DDC/TDC (aromatic-L-amino-acid/L-tryptophan decarboxylase), L-tryptophan is decarboxylated to form tryptamine, which is subsequently condensed with secologanin by STR1 (strictosidine synthase) to yield 3-α(S)-strictosidine. This intermediate is then hydrolyzed by SGR1 (strictosidine beta-D-glucosidase) to produce strictosamide, followed by sequential enzymatic modifications leading to vellosimine. The final step involves oxidation catalyzed by vellosimine dehydrogenase to generate tombozine.

Transcriptomic analysis identified 13 differentially expressed genes (DEGs) associated with this pathway l (**Figure 7B**), including 5 DDC/TDC isoforms, 7 STR1 homologs, and 1 vellosimine dehydrogenase gene. These DEGs exhibited distinct expression patterns across three *Murraya* species (*M. exotica*, *M. tetramera*, and *M. kwangsiensi*). Notably, the expression profile of BMK_Unigene_186409 (annotated as DDC/TDC) showed striking consistency with tombozine accumulation trends, suggesting its potential regulatory role in the species-specific differential biosynthesis of tombozine.

## Discussion

*Murraya*, a traditional ethnomedicinal plant, is widely used in folk medicine, with extensive studies confirming its chemical diversity and pharmacological potential. Continued exploration using advanced methodologies is expected to further contribute to human health. Alkaloids, as key bioactive metabolites in plants, show notable interspecies variation in composition and bioactivity. Previous studies have reported alkaloid diversity across species: 44 alkaloids with distinct accumulation patterns during fruit development in *Ziziphus jujuba* and *Ziziphus* sp*inosa* (Xue et al., 2023); 44 differentially accumulated alkaloids in four cultivars of *Heuchera micrantha* related to environmental adaptation (Gong et al., 2024); 81 material-specific alkaloids in *Houttuynia cordata* (Jiang et al., 2024); and 64 light-responsive alkaloids in *Catharanthus roseus* (Nagy et al., 2023). These findings highlight the taxonomic and functional diversity of plant alkaloids. In this study, we identified 77 alkaloid metabolites with species-specific accumulation in three *Murraya* species (*M. exotica*, *M. tetramera*, and *M. kwangsiensis*). Notably, our results differ from a prior report detecting only 19 alkaloids in *M. tetramera* leaves (Zhou et al., 2024), a discrepancy possibly due to geographic variations and methodological differences. This underscores the need for systematic studies on alkaloid accumulation in *Murraya* to optimize pharmaceutical applications.

Alkaloid metabolites exhibit diverse pharmacological effects, particularly antitumor activity. Vinblastine and vincristine from *Catharanthus roseus* inhibit microtubule polymerization, blocking mitosis and inducing apoptosis, while suppressing telomerase to limit proliferation (Yamamoto et al., 2016; Li et al., 2025). Paclitaxel from Taxus stabilizes microtubules, arresting cells at G2/M phase (Kim et al., 2016). Other alkaloids, including triptolide (Xiao et al., 2024), camptothecin (Zhang et al., 2023), homoharringtonine (Xi et al., 2024), podophyllotoxin (Motyka et al., 2023), and colchicine (Hawash, 2022), act through multi-target mechanisms involving cell cycle modulation, apoptosis, and angiogenesis inhibition.

Carbazole alkaloids from *Murraya* show promising antitumor properties. Mahanimbine induces apoptosis and cell cycle arrest; in HL-60 cells, it triggers G2/M arrest and caspase-3 activation (Hobani, 2022). It also suppresses migration and invasion in A-549 lung cancer cells, indicating potential against metastasis.

Through network pharmacology and molecular docking, this study identified three key alkaloids, including aegeline, tombozine, and crotaleschenine that stably bind to oncogenic targets (PIK3CD, MAPK8, PIK3CA, and JAK2). Functional enrichment revealed their roles in regulating tumor proliferation and apoptosis via PI3K/Akt and MAPK pathways. These alkaloids may also modulate immune responses, such as suppressing virus-induced inflammation, to inhibit herpesvirus-linked tumorigenesis. Together, *Murraya* alkaloids demonstrate multi-dimensional therapeutic potential with significant value in oncology.

Alkaloid biosynthesis involves pathways like indole, isoquinoline, and tropane/piperidine/pyridine alkaloid synthesis, intersecting with amino acid metabolism (Gong et al., 2024). Key enzymes, such as DDC/TDC, catalyze decarboxylation reactions critical for alkaloid production. In *Catharanthus roseus*, tryptophan decarboxylase (TDC) converts tryptophan to tryptamine, a key step in monoterpenoid indole alkaloid (MIA) biosynthesis. Tryptamine then condenses with secologanin via strictosidine synthase (STR) to form strictosidine, the precursor of vinblastine and vincristine. *TDC* expression in laticifers and idioblasts correlates with alkaloid synthesis sites (Yamamoto et al., 2016; Li et al., 2024). Metabolic engineering of *TDC* has increased tryptamine flux and alkaloid yields.

Similarly, *TDC* initiates camptothecin biosynthesis in *Camptotheca acuminata* (Qiao et al., 2022). Here, transcriptomic analysis outlined the tombozine pathway in *Murraya*, identifying a *DDC*/*TDC* homolog linked to tombozine accumulation. Comparative genomics revealed that genetic factors, including gene regulation, gene family evolution, and enzyme kinetics underpin species-specific alkaloid profiles in *M. exotica*, *M. tetramera*, and *M. kwangsiensis*. These insights demonstrate how genomic adaptation shapes metabolic diversity, offering targets for engineering alkaloid biosynthesis.

While this study provides critical insights into *Murraya* alkaloids, several limitations remain. First, the sample size and geographic scope were restricted, potentially overlooking environmental influences on alkaloid variability. Second, the functional validation of candidate genes in alkaloid biosynthesis requires further experimental confirmation. Third, the pharmacological evaluation relied on computational predictions, necessitating *in vitro* and *in vivo* assays to verify efficacy and safety.

Future research should expand to additional *Murraya* species and populations to capture broader phytochemical diversity. Mechanistic studies, including CRISPR-based gene editing and heterologous expression, could elucidate biosynthetic pathways and regulatory networks. Clinical trials are essential to translate computational findings into therapeutic applications, particularly for cancer and inflammatory diseases. Integrating synthetic biology approaches may enable scalable production of high-value alkaloids, fostering sustainable exploitation of medicinal plant resources. Addressing these gaps will enhance the scientific and industrial impact of *Murraya* alkaloid research.

## Conclusion

This study systematically deciphered the alkaloid composition, pharmacological relevance, and biosynthetic regulation in three *Murraya* species. The identification of 77 alkaloids, including species-specific metabolites, highlights the genus’ chemical diversity and ecological adaptability. Network pharmacology and molecular docking revealed that core alkaloids (tombozine, aegeline, and crotaleschenine) target key oncogenic pathways (PI3K/Akt and MAPK), offering insights into their antitumor and anti-inflammatory mechanisms. Notably, *M. kwangsiensis* exhibited superior accumulation of pharmacologically active alkaloids, suggesting its enhanced medicinal potential. Transcriptomic insights into tombozine biosynthesis further linked enzymatic regulation (DDC/TDC expression) to species-specific metabolic divergence. Collectively, this work bridges phytochemical discovery with biosynthetic enzymology, advancing the translational potential of *Murraya* alkaloids in drug development and ethnopharmacological modernization.

## Data Availability

The datasets presented in this study can be found in online repositories. The names of the repository/repositories and accession number(s) can be found below: https://www.ncbi.nlm.nih.gov/, PRJNA1218029.

## Supplementary material

Supplementary Table 1 PCA variance proportion.

Supplementary Figure 1 The Venn diagram of differential alkaloid metabolites in different comparison groups.
