# Supplementary figures and images for: Metabolic profiling and pharmacological evaluation of alkaloids in three Murraya species
Source: Front Plant Sci. 2025 Oct 15;16:1675533. doi: 10.3389/fpls.2025.1675533 (PMC12568637; doi:10.3389/fpls.2025.1675533)

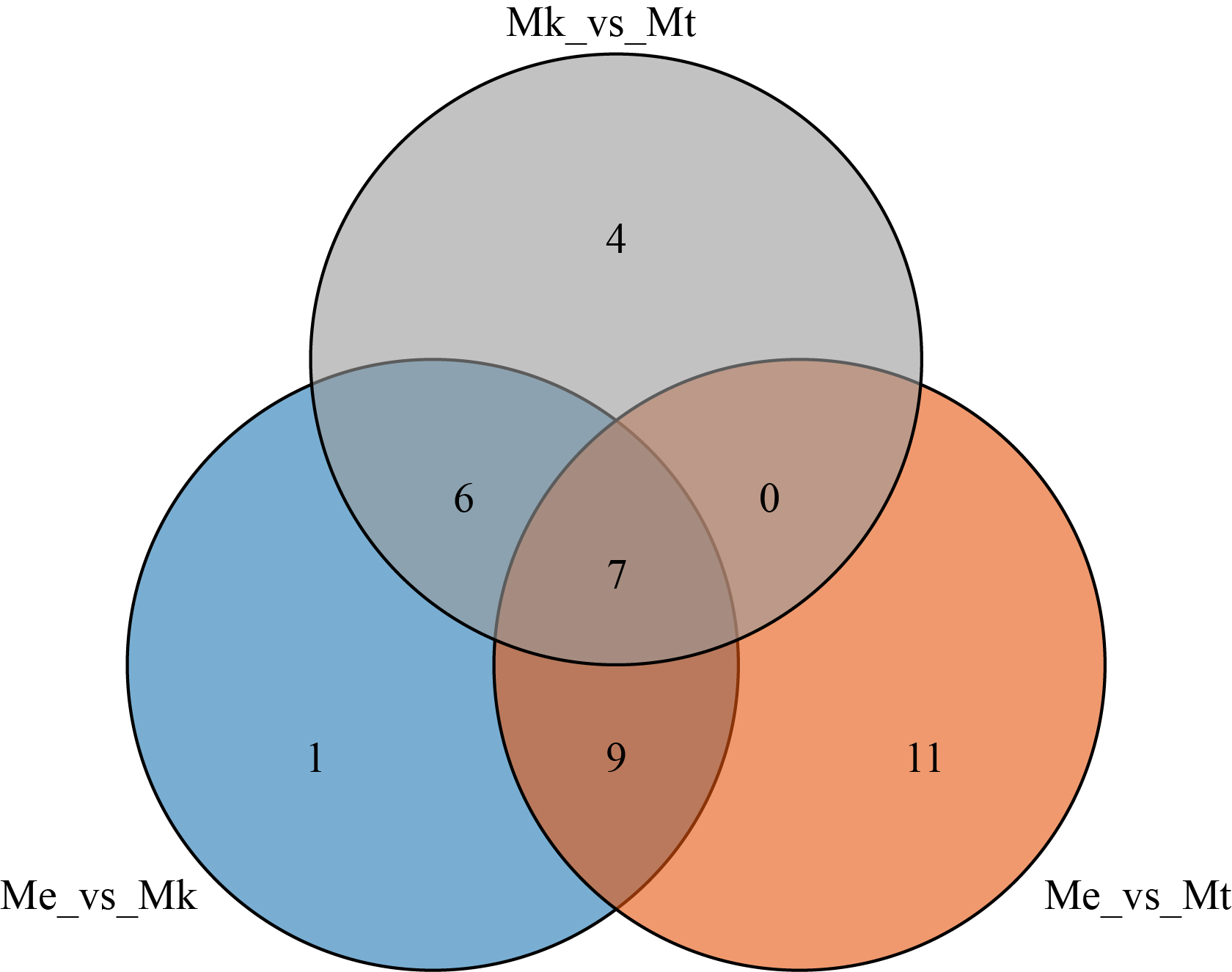

Supplement: Supplementary Figure 1 — The Venn diagram of differential alkaloid metabolites in different comparison groups. [file Image1.jpeg]
